# Supplementary material for: The cost effectiveness of early assessment and intervention by a dedicated health and social care professional team for older adults in the emergency department compared to treatment-as-usual: Economic evaluation of the OPTI-MEND trial
Source: PLoS One. 2024 Jun 25;19(6):e0298162. doi: 10.1371/journal.pone.0298162 (PMC11198796; doi:10.1371/journal.pone.0298162)
Supplement: S1 File — (DOCX) [file pone.0298162.s001.docx]

**Supplementary Materials**

**Appendix 1: Cost of the dedicated HSCP team for the trial period**

Supplementary Table 1 presents the total budget to provide early assessment and intervention to older adults in the Emergency Department (ED) by a dedicated HSCP team in the OPTI-MEND study.

**Supplementary Table 1: Budget allocated to provide dedicated HSCP team in the OPTI-MEND trial.**

| **Role** | **FTE** | **Gross Salary** | **6 Months** | **PRSI** | **Pension** | **Total** |
| --- | --- | --- | --- | --- | --- | --- |
| Senior Physiotherapist (HSE scale-mid scale) | 1 | €57.687,00 | €28.843,50 | €3.187,21 | €5.768,70 | €37.799,41 |
| Senior Occupational Therapist (HSE scale -mid scale) | 1 | €57.687,00 | €28.843,50 | €3.187,21 | €5.768,70 | €37.799,41 |
| Senior Medical Social worker (HSE Scale -mid scale) | 1 | €65.920,00 | €32.960,00 | €3.642,08 | €6.592,00 | €43.194,08 |
| Space | NA | €29.200,00 | €14.600,00 | - | - | €14.600,00 |
| Equipment | NA | €15.000,00 | €7.500,00 | - | - | €7.500,00 |
| *FTE: Full Time Equivalent, PRSI: Pay Related Social Insurance.*  *Equipment costs include mobility aids and activities of daily living aids/appliances for HSCP participants. Space costs relate to a dedicated assessment room for the HSCP team in the ED.* | | | | **Total budget to provide HSCP** | | **€140.892,89** |

Supplementary Table 2 provides the mean incremental cost of early HSCP assessment and intervention per participant (n=176) allocated to the HSCP arm and indicates the average cost per participant to be €800.53.

Supplementary Table 2: Calculation of the average cost per participant who received HSCP in addition to treatment as usual.

| **Item** | **values** | **Source** |
| --- | --- | --- |
| Total budget for adding HSCP in the ED | €140.892,89 | Supplementary Table 1 |
| Number of participants who received HSCP in OPTI-MEND trial | 176 | OPTI-MEND Trial |
| Average cost per participant to receive HSCP in addtion to TAU | €800.53 | (Total budget for adding HSCP in the ED) / ( Number of participant who received HSCP in OPTI-MEND trial) |

**Appendix 2: Cost of the Emergency Department admission provided by University Hospital of Limerick for 2020**

Supplementary Table 3 is a summary of data purcured from the finance department of University of Limerick Hospital to determine the annual running cost in 2020.

**Supplementary Table 3: Annual budget of the Emergency Department, University Hospital of Limerick (2020)**

| **Class** | **YTD Actual** | **Source** |
| --- | --- | --- |
| Pay | 15,527,158 | Data on File. Department of Finance, University Hospital Limerick |
| Non Pay | 3,369,709 | Data on File. Department of Finance, University Hospital Limerick |
| Total annual budget | 18,896,867 | Data on File. Department of Finance, University Hospital Limerick |

Based on the total annual running cost of the ED, Supplementary Table 4 calcuates the average cost per patient visiting ED. Furthermore, by referring to the statiistics observed on average length of stay under Treatment as Usual (TAU) in the OPTIMEND trial, we estimate average cost per hour spent in the ED.

| **Parameter name** | **Value** | **Source** |
| --- | --- | --- |
| Total annunal budget to run ED | €18,896,867 | Supplementary Table 3 |
| Annual number of adult patients presenting in the ED | 71,315 | <https://healthservice.hse.ie/filelibrary/ulh/ul-hospitals-group-annual-review-2019.pdf>, page 6 |
| Mean Cost per ED visit | € 264.98 | (Total annual budget) / (Annual number of patients presenting in the ED) |
| Average number of hours spent in ED | 18.11 | Summary statistic, OPTIMEND trial (TAU arm) |
| Average number of hours spent in ED | 11.50 | Summary statistic, OPTIMEND trial (HSCP arm) |
| Average cost per hour spent in the ED | €14.63 | (Mean Cost per Patient) / (Average number of hours spending in ED (Observed in TAU arm)) |

**Supplementary Table 4::Calculations of the average cost of ED visit and average cost per hour spent in ED.**
